# Identification of Genes Associated with the Metastasis of Pheochromocytoma/Paraganglioma Based on Weighted Gene Coexpression Network Analysis

**DOI:** 10.1155/2020/3876834

**Published:** 2020-02-05

**Authors:** Qisheng Su, Qinpei Ding, Zunni Zhang, Zheng Yang, Yuling Qiu, Xiaohong Li, Wuning Mo

**Affiliations:** ^1^Department of Clinical Laboratory, First Affiliated Hospital of Guangxi Medical University, Nanning, Guangxi Zhuang Autonomous Region, China; ^2^Department of Endocrinology, First Affiliated Hospital of Guangxi Medical University, Nanning, Guangxi Zhuang Autonomous Region, China

## Abstract

**Background:**

Pheochromocytoma/paraganglioma (PCPG) is a benign neuroendocrine neoplasm in most cases, but metastasis and other malignant behaviors can be observed in this tumor. The aim of this study was to identify genes associated with the metastasis of PCPG.

**Methods:**

The Cancer Genome Atlas (TCGA) expression profile data and clinical information were downloaded from the cbioportal, and the weighted gene coexpression network analysis (WGCNA) was conducted. The gene coexpression modules were extracted from the network through the WGCNA package of R software. We further extracted metastasis-related modules of PCPG. Enrichment analysis of Biological Process of Gene Ontology and Kyoto Encyclopedia of Genes and Genomes was carried out for important modules, and survival analysis of hub genes in the modules was performed.

**Results:**

A total of 168 PCPG samples were included in this study. The weighted gene coexpression network was constructed with 5125 genes of the top 25% variance among the 20501 genes obtained from the database. We identified 11 coexpression modules, among which the salmon module was associated with the age of PCPG patients at diagnosis, metastasis, and malignancy of the tumors.

**Conclusion:**

WGCNA was performed to identify the gene coexpression modules and hub genes in the metastasis-related gene module of PCPG. The findings in this study provide a new clue for further study of the mechanisms underlying the PCPG metastasis.

## 1. Introduction

Pheochromocytoma/paraganglioma (PCPG) is a neoplasm that synthesizes and secretes catecholamines (CA) in the neuroendocrine system. It refers to pheochromocytoma as the tumor occurs in an adrenal gland, whereas it is termed paraganglioma when the neoplasm arises in extra-adrenal tissues. PCPG can lead to elevated blood pressure in patients, thus causing damage to target organs such as the heart, brain, and kidney. Typically, PCPG features paroxysmal hypertension with palpitations, hyperhidrosis, and headache. Also, it can cause disorders in blood sugar and insulin metabolism, thereby endangering life [1, 2]. PCPGs are mostly benign tumors with a potential for malignant transformation; some cases could develop into malignant tumors [3]. Metastasis is the main manifestation of PCPG malignant transformation. At present, surgical resection is the main way to treat PCPG, yet therapeutic regimen and efficacy are closely related to the metastasis status of PCPG [3].

In recent years, genetic changes in PCPG have received increasing attention, and mutations or loss of genes encoding succinate dehydrogenase (SDH) are clinically important genetic changes [4, 5], which are associated with the increased metastatic rate of human PCPG. It has been proved that the pathogenesis of nonfamilial PCPG involves *SDHB* mutation-associated PCPG pro-cadherin *γ*-C3 (*PCDHGC3*) gene promoter methylation [6] as well as *EPAS1* mutations encoding HIF2*α* [7]. Given that those genetic changes play important roles in the development of PCPG, identification of the genes with a key role in PCPG could be beneficial to the diagnosis, treatment, and prognostic prediction of this neoplasm. Thus, the exploration of new genes related to the clinical traits of PCPG will facilitate the current diagnosis and treatment of PCPG.

Weighted gene coexpression network analysis (WGCNA) is a widely used method in biology and medical research, which aims at mining module information from chip or sequencing data [8]. In this method, modules are defined as a group of genes with similar expression patterns. Genes with the same expression patterns may usually participate in common biological processes and pathways. Associating coexpression gene modules with phenotypic traits can infer the biological processes and pathways related to disease phenotypes and explore the pathogenesis of disease. In this study, we used the WGCNA method to construct gene coexpression network, identify gene modules related to clinical phenotypes of PCPG's malignancy and metastasis, and screen hub genes in the module. The findings could provide a basis for further study of PCPG and its molecular targeted therapy.

## 2. Materials and Methods

### 2.1. Expression Profile Data of Pheochromocytoma/Paraganglioma

The expression profile data normalized by the median and clinical information of PCPG in the Cancer Genome Atlas (TCGA) were obtained from cbioportal [9] (http://www.cbioportal.org/). The information regarding clinical staging, metastasis, and malignancy of the tumors was available for this study. A total of 168 PCPG cases with complete clinical parameters were included in the present study.

### 2.2. Analysis of Gene Coexpression Modules

*R* (version 3.5.3) was used for the data analysis. The expression data were screened by variance analysis. Genes with the top 25% variance of expression values among samples were determined. WGCNA was performed to study the gene coexpression modules and their clinical characteristics related to PCPG. The Pearson correlation coefficient between every two genes was calculated, and the correlation value was determined by using the soft thresholding power available from picksoftthreshold function of the WGCNA package. Dynamic tree cut algorithm was employed to identify gene coexpression modules. The minimum module size of 30 and the merged cutting height of 0.25 were set. Cytoscape 3.6.1 was used to map the gene coexpression network of important modules.

### 2.3. Clinical Traits-Based Module-Trait Analysis

Information regarding gender, age at diagnosis, distant metastasis, recurrence, and malignancy of PCPG was obtained from cbioportal. Pearson correlation coefficients between modules and clinical traits were calculated, and modules significantly correlated with individual traits were determined (*P* < 0.05). Genes in those positive modules were then subjected to further analysis.

### 2.4. Biological Process and Pathway Enrichment Analysis

Genes in key modules were submitted to Webgestalt (http://www.webgestalt.org/), a web tool for functional enrichment analysis [10]. Enrichment analysis of biological processes of Gene Ontology (GO) and Kyoto Encyclopedia of Genes and Genomes (KEGG) pathways was then carried out by using Fisher's exact test, and the *P* value was adjusted by the Benjamin–Hochberg method.

### 2.5. Screening of Hub Genes and Survival Analysis

Starbase (http://starbase.sysu.edu.cn/) is a comprehensive pan-cancer analysis platform generated in the Qu Lab, Sun Yat-sen University of China [11], and Pan-Cancer (http://starbase.sysu.edu.cn/pancer.php) of Starbase is a TCGA-based tool. In this study, genes with high gene significance and module correlation in important modules were considered as hub genes. An absolute value of module membership (MM) > 0.8 and absolute value of gene significance (GS) in significant clinical traits > 0.2 were defined as screening criteria for hub genes in the key module identified in the present study. According to the mean value of gene expression, TCGA samples in Pan-cancer tool of Starbase were divided into two groups: high-expression group and low-expression group. The effects of hub genes on the survival of PCPG patients were analyzed based on the Kaplan–Meier method via Pan-cancer tool.

## 3. Results

### 3.1. Identification of Gene Coexpression Modules

**Figure 1:**
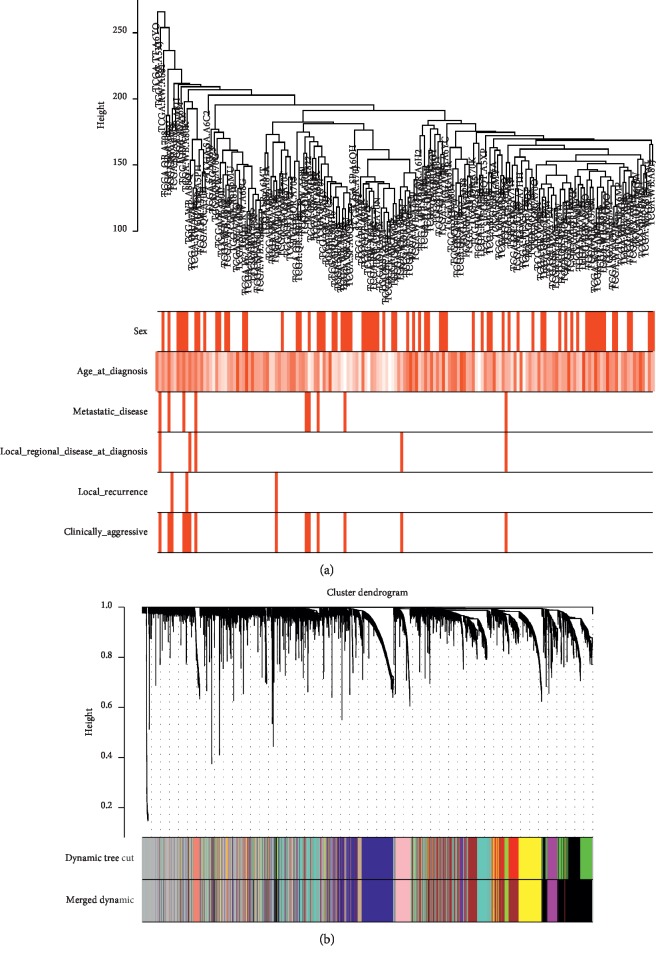
Gene coexpression module analysis. (a) Sample dendrogram and trait heatmap. (b) Gene dendrogram and identified coexpression modules.

A total of 20501 genes with the expression profile data were obtained, and 515 genes from the top 25% variance were included in the WGCNA. In order to ensure scale-free network, we chose a soft threshold of *β* = 6 with a minimum module size of 30. A coexpression gene network was constructed. The adjacency matrix was transformed into a topological overlap matrix, and a hierarchical clustering tree was generated. As shown in Figure 1, when cutting height was set to 0.25, the WGCNA identified 11 gene coexpression modules, while no modules were merged.

### 3.2. Module-Trait Relationship Analysis

**Figure 2:**
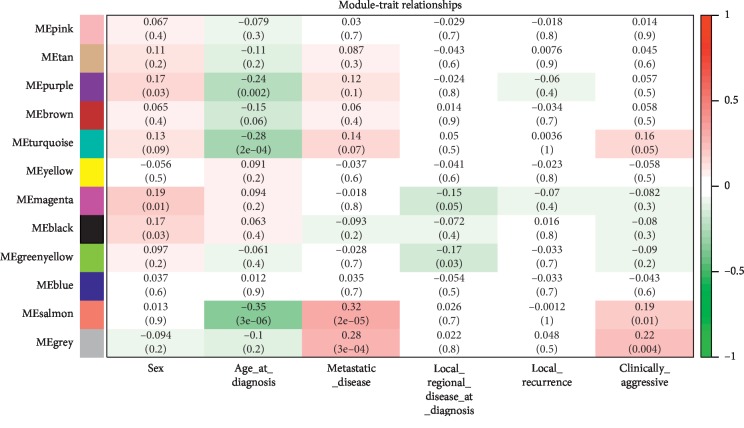
Module-trait relationships. ME means the module eigengene. Red blocks and green ones indicated positive correlation and negative correlation between ME and the trait, respectively. Color depth was proportional to the absolute value of correlation coefficient (the upper number and lower number in each color block represented the Pearson correlation coefficient and *P* value, respectively).

Module-trait relationship analysis revealed that Module Eigengenes (MEs) of the salmon module were highly correlated with age, metastasis, and malignancy of PCPG patients (*P* < 0.05), but not with gender and in situ recurrence of the cases (*P* > 0.05) (Figure 2).

### 3.3. Gene Ontology and KEGG Pathway Enrichment Analysis

**Figure 3:**
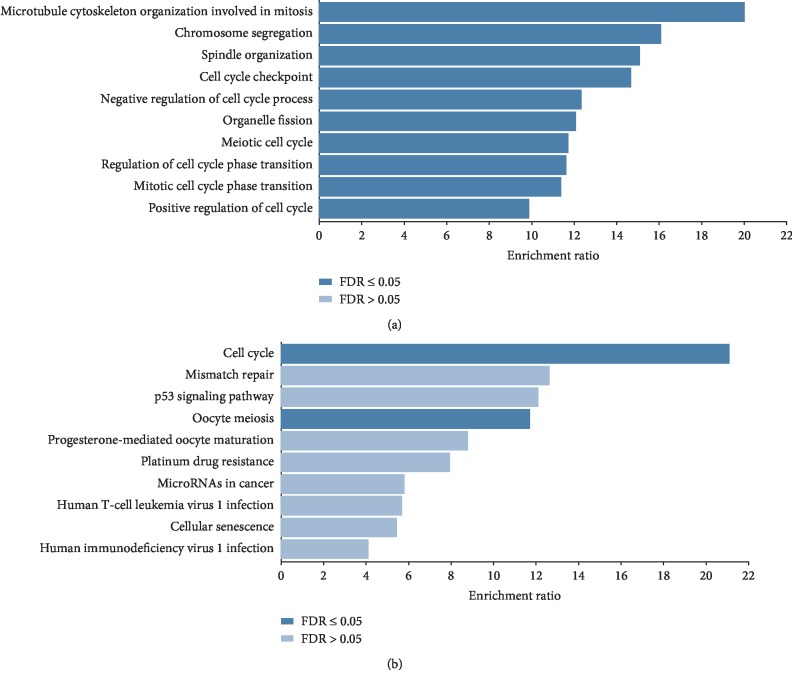
Biological processes of Gene Ontology (a) and Kyoto Encyclopedia of Genes and Genomes enrichment analysis based on Webgestalt (b).

All genes in the salmon module were submitted to Webgestalt for GO biological processes and KEGG pathway enrichment analysis. As depicted in Figure 3(a), salmon module genes were enriched in mitotic cell cycle phase transition, organelle fission, chromosome segregation, and other biological processes related to cell division. In the meantime, KEGG pathway enrichment analysis revealed that genes in this module were enriched in cell cycle and oocyte meiosis pathway (Figure 3(b)).

### 3.4. Survival Analysis of Hub Genes

**Figure 4:**
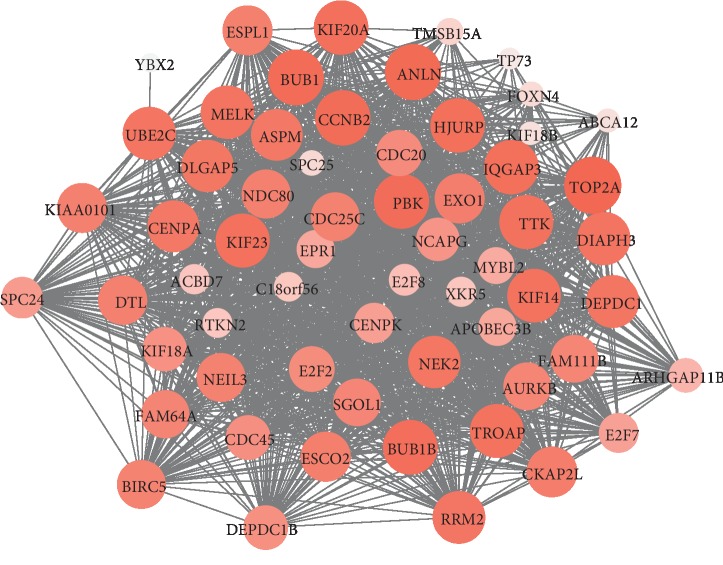
Gene coexpression network of the salmon module. The size and color intensity in each circle were proportional to the absolute value of module membership (MM).

**Figure 5:**
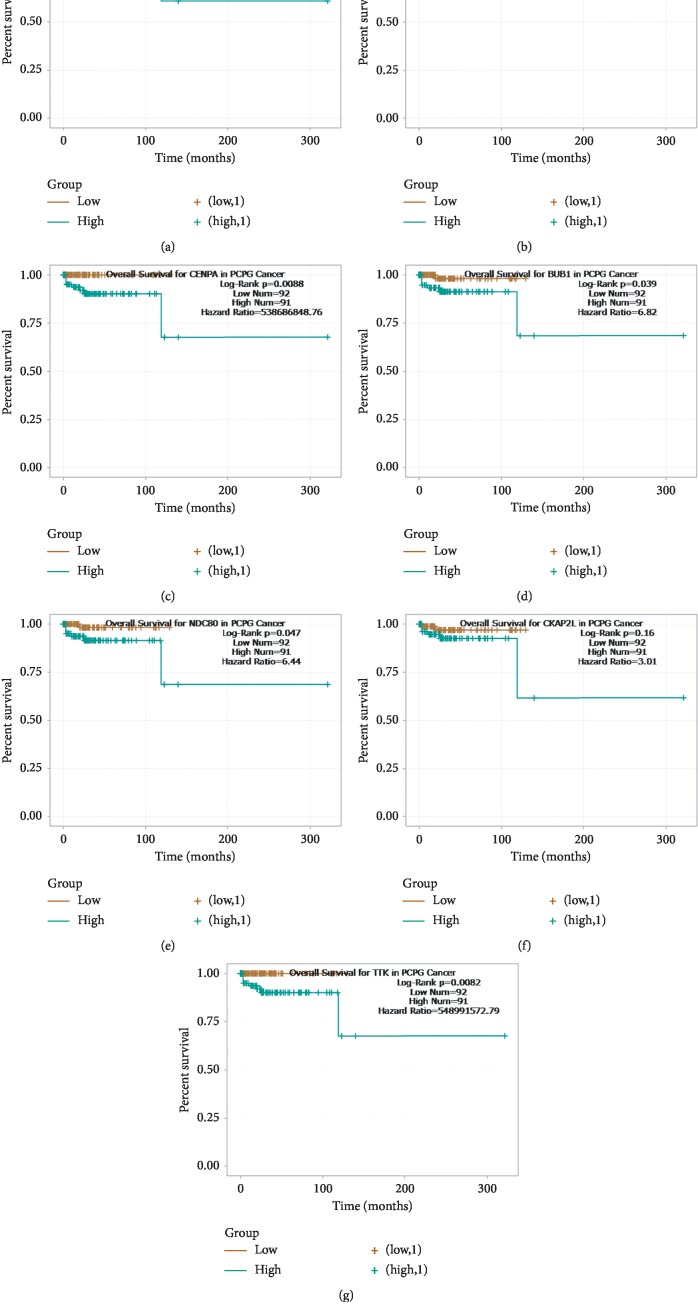
Survival analysis of hub genes in the salmon module for PCPG patients.

Based on the screening criteria (|MM| > 0.8 and |GS| > 0.2), we identified seven hub genes in the salmon module, including *TOP2A*, *BUB1*, *BUB1B*, *TTK*, *CENPA*, *NDC80*, and *CKAP2L* (Figure 4, Table 1). As depicted in Figure 5, the survival analysis of these hub genes by using Starbase database revealed that PCPG patients with a high expression of *BUB1*, *BUB1B*, *TTK*, *CENPA*, or *NDC80* had shorter survival time and higher risk of death (*P* < 0.05 and hazard ratio >1). Meanwhile, we observed *P* values greater than 0.05, but high hazard ratios in the cases of *CKAP2L* and *TOP2A*, suggesting that these two genes could be potential risk factors for PCPG.

## 4. Discussion

As a malignant behavior, tumor metastasis is an important factor in determining the treatment regimen of PCPG. Thus, studies on PCPG metastasis will contribute to further analysis of the treatment efficacy and prognosis of this malignancy. In this study, we performed the WGCNA on the expression profiles of PCPG patients in TCGA and identified 11 gene coexpression modules. We further showed that the patients with a high expression of genes in the salmon module had an earlier onset age, and the eigengene of the salmon module was highly correlated with the metastasis as well as malignancy of tumors. GO enrichment analysis revealed that salmon module genes were significantly enriched in mitotic cell cycle phase transition, organelle fission, chromosome segregation, and other cell division-related biological processes. Meanwhile, KEGG analysis demonstrated that genes in the salmon module were highly enriched in cell cycle and oocyte meiosis pathways. Notably, the above processes and pathways mainly involve cell proliferation, which may play an important role in the occurrence and development of PCPG. Furthermore, GS- and MM-based screening led us to identification of seven hub genes for PCPG, including *TOP2A*, *BUB1*, *BUB1B*, *TTK*, *CENPA*, *NDC80*, and *CKAP2L*. In fact, these hub genes encode the essential enzymes and components for cell division and proliferation, providing consistent data for the GO and KEGG enrichment analysis in this study. Although the salmon module was found to be related to the tumor metastasis in PCPG, GO and KEGG enrichment analysis did not reveal biological processes and pathways related to cell invasion and movement. In this case, we speculate that salmon module genes may promote tumor cell metastasis by regulating the proliferation and division of cancer cells. Besides, Starbase platform-based survival analysis indicated that the survival time of PCPG patients with high expression of the hub genes was significantly shorter, and the hazard ratio for each group analyzed was greater than 1. These data showed that the high expression of the hub genes was associated with poor prognosis of PCPG patients, suggesting that the hub gene expression acts as a potential prognostic indicator for PCPG. Given that poor prognosis and death of cancer patients can be attributed to tumor cell proliferation and metastasis, survival analysis of the hub genes in this study obtained consistent data with the WGCNA as well as salmon module-trait relationship analysis.

Among hub genes in the salmon module, *TOP2A* encodes DNA topoisomerase 2A that controls and alters DNA topology during transcription, thus participating in chromosome concentration, chromatid isolation, and DNA transcription and replication [12]. *TOP2A* is currently the target of many anticancer drugs. Immunohistochemical studies of PCPG showed that the expression of *TOP2A* in malignant lesions was significantly higher than that in benign lesions [13]. The proteins encoded by genes *BUB1*, *BUB1B*, and *TTK* have the ability to phosphorylate serine and threonine residues in the substrates [14], while *TTK* can also phosphorylate tyrosine residues, acting on checkpoints during cell mitosis [15]. It has been found that numerous tumors, such as glioblastoma [16, 17] and gastric adenocarcinoma [18], harbor expression changes in *BUB1*, *BUB1B*, and *TTK* as well as checkpoint dysfunction. Moreover, *BUB1*, *BUB1B*, and *TTK* were shown to be associated with malignant behaviors such as PCPG metastasis in TCGA-based studies [19, 20]. The above observation was consistent with the findings in the present study. While *CENPA* is a member of the histone H3 family, *NDC80* encodes a kinetochore component. Prior to the present study on *CENPA* and *NDC80*, multiple studies have reported that these two factors were associated with poor prognosis of patients with lung adenocarcinoma, osteosarcoma, or other tumors [21–23].

Studies on *CKAP2L* are currently focused on Filippi syndrome [24, 25], rather than tumors. The high expression of *CKAP2L* in lung adenocarcinoma was found to be associated with the tumor stage, lymph node status, and metastasis. It has been reported that *CKAP2L* may act on the proliferation, migration, and invasion of lung adenocarcinoma cells through mitogen-activated protein kinase (MAPK) signaling pathway [26], but its role in other tumors has yet to be fully studied. To our knowledge, the present study provided the first demonstration that *CKAP2L* may be related to PCPG metastasis. However, its molecular function and role in cancer need to be further clarified.

## 5. Conclusions

The aim of this study was to identify genes associated with clinical traits of PCPG by WGCNA. We found that the salmon module is associated with PCPG metastasis. Genes in this module were rarely studied in PCPG. Further studies on these genes will contribute to a better understanding of the PCPG metastasis process as well as facilitating the clinical treatment and prognosis analysis of PCPG. However, it remains to be determined whether these genes could be new drug targets for PCPG treatment. There were still some limitations in this study. More traits could not be included in the study, due to limited available clinical information. We will conduct experimental studies on how these hub genes function in PCPG metastasis through manipulating their expression in cells.

## Figures and Tables

**Table 1:** Module membership (MM) and gene significance (GS) of significant clinical traits of hub genes in the salmon module.

Gene	GS of age at diagnosis	GS of metastatic disease	GS of clinically aggressive tumors	MM of the salmon module
*BUB1B*	−0.30346	0.36041	0.242502	0.912711
*TOP2A*	−0.37821	0.334621	0.220242	0.947308
*CENPA*	−0.30716	0.309183	0.215869	0.868744
*BUB1*	−0.29943	0.344204	0.210993	0.927818
*NDC80*	−0.41786	0.253726	0.210538	0.827401
*CKAP2L*	−0.27829	0.28499	0.202205	0.848142
*TTK*	−0.34046	0.275445	0.200101	0.90299

## Data Availability

The data used in this study were obtained from open public databases, and data acquisition is explained in the manuscript.
